# Machine learning approach to determine the diagnostic value and predictive factors of PET/CT in FUO and IUO patients

**DOI:** 10.3389/fmed.2026.1763501

**Published:** 2026-03-16

**Authors:** Sule Ceylan, Bahadir Ceylan, Oktay Olmuscelik, Tansel Cakir

**Affiliations:** 1 Department of Nuclear Medicine, University of Health Science, Gaziosmanpasa Training and Research Hospital, Istanbul, Türkiye; 2 Department of Infectious Diseases and Clinical Microbiology, Medical Faculty, Istanbul Medipol University, Istanbul, Türkiye; 3 Department of Internal Medicine, Medical Faculty, Istanbul Medipol University, Istanbul, Türkiye; 4 Department of Nuclear Medicine, Medical Faculty, Istanbul Medipol University, Istanbul, Türkiye

**Keywords:** diagnostic prediction, fever of unknown origin, inflammation of unknown origin, machine learning, PET/CT

## Abstract

**Objective:**

This study aimed to identify the clinical and laboratory variables determining the true diagnostic contribution of FDG-PET/CT in patients with fever of unknown origin (FUO) or inflammation of unknown origin (IUO), and to develop machine learning models capable of predicting which patients are most likely to benefit from PET/CT imaging.

**Methods:**

A retrospective cohort of patients aged over 18 years who underwent FDG-PET/CT for FUO/IUO evaluation was analyzed. Machine learning algorithms—including Extreme Gradient Boosting (XGBoost), linear and radial basis function Support Vector Machines, Multilayer Perceptron (MLP), k-Nearest Neighbors (KNN), Random Forest, Decision Tree, Logistic Regression (LR), and Naïve Bayes (NB)—were trained to predict true positive and true negative PET/CT results. Feature selection was performed using the PowerSHAP method. Model performance was compared using area under the precision–recall curve (PR-AUC), Receiver Operating Characteristic – Area Under the Curve (ROC-AUC), accuracy, precision, recall, and F1-score metrics.

**Results:**

A total of 273 patients (151 men, 55.3%; mean age: 59 ± 16.9 years) were included. PET/CT provided diagnostic benefit in 203 patients (74.4%). All algorithms performed well in terms of PR-AUC (>0.79), with the highest scores achieved by MLP and XGBoost, reaching PR-AUC values of 0.86 and 0.85, respectively. All algorithms except NB demonstrated good accuracy. When both PR-AUC and accuracy were considered together, the best-performing models were MLP and Logistic Regression. LR achieved accuracy, ROC-AUC, PR-AUC, precision, recall, and F1-score values of 0.75, 0.74, 0.84, 0.85, 0.86, and 0.83, respectively, whereas MLP achieved 0.73, 0.73, 0.86, 0.85, 1.00, and 0.85. The PowerSHAP analysis suggested that lower procalcitonin and erythrocyte sedimentation rate levels, longer symptom duration, older age, generalized body pain, inpatient evaluation, and higher lymphocyte counts were associated with increased model-predicted PET/CT usefulness.

**Conclusion:**

Machine learning models—particularly MLP and LR—may have potential to assist in identifying FUO/IUO patients who could benefit from PET/CT imaging. The clinical and biochemical predictors highlighted in this study might help guide PET/CT use and support more tailored diagnostic approaches in complex FUO and IUO cases, though further validation is needed.

## Introduction

Fever of Unknown Origin (FUO) and Inflammation of Unknown Origin (IUO) represent overlapping clinical entities characterized by prolonged inflammation without an identifiable cause. FUO is classically defined as a body temperature exceeding 38.3 °C on multiple occasions for more than 3 weeks, with the etiology remaining undetermined after at least 1 week of appropriate investigation (1). In contrast, IUO describes patients with persistent systemic inflammation and elevated inflammatory markers in the absence of fever (2). Both conditions pose considerable diagnostic challenges due to their broad and heterogeneous differential diagnoses, encompassing infectious, malignant, autoimmune, and miscellaneous causes.

Positron emission tomography with fluorine-18-fluorodeoxy glucose combined with computed tomography (FDG-PET/CT) has emerged as a valuable diagnostic modality in the evaluation of patients with FUO and IUO (3–13). By detecting regions of increased metabolic activity, PET/CT can uncover infectious, inflammatory, or neoplastic foci that may remain undetected on conventional imaging techniques. Previous studies have shown that PET/CT can significantly aid in establishing a diagnosis in a substantial proportion of cases.

Nevertheless, PET/CT is not without limitations. The technique involves exposure to ionizing radiation and incurs considerable financial cost. Furthermore, in a subset of patients, PET/CT findings may be inconclusive or misleading, potentially resulting in unnecessary diagnostic procedures or interventions (14). Therefore, it is crucial to determine which patients are most likely to benefit from PET/CT imaging to optimize its clinical utility.

In the literature, only a limited number of studies have examined the diagnostic contribution of PET/CT and the clinical or laboratory variables associated with its usefulness in patients with FUO or IUO (3–13). Moreover, except for two investigations (7, 9), most studies have included relatively small patient cohorts, limiting the generalizability of their findings. Differences in sample size, variable selection, and the definition of PET/CT usefulness have further contributed to inconsistent and sometimes contradictory results regarding the predictors of PET/CT benefit.

To date, no study has applied machine learning (ML)–based methods to predict the diagnostic yield or necessity of PET/CT in FUO or IUO. ML provides a novel, data-driven analytical framework capable of capturing complex interactions among demographic, clinical, and laboratory parameters to identify meaningful predictive patterns.

Accordingly, the present study aimed to identify the variables determining the true diagnostic contribution of PET/CT in patients with FUO or IUO and to develop machine learning models capable of predicting which patients are most likely to benefit from PET/CT imaging.

## Materials and methods

This study was designed as a retrospective cohort study. It included patients who were evaluated for FUO or IUO at Istanbul Medipol University between 2015 and 2016.

### Inclusion criteria

- Fulfillment of the diagnostic criteria for FUO or IUO.
- Age over 18 years.
- Undergoing PET/CT imaging as part of the diagnostic work-up.

### Exclusion criteria

- Patients who did not meet the diagnostic criteria for FUO or IUO.

### PET/CT protocol

All patients underwent whole-body 18F-FDG PET/CT imaging using a Siemens Biograph mCT scanner. Prior to tracer injection, patients fasted for at least 6 hours, and blood glucose levels were confirmed to be below 150 mg/dL. An intravenous dose of 0.1 mCi/kg (3.7 MBq/kg) of 18F-FDG was administered, followed by a 60-min uptake period in a quiet, dimly lit room to minimize muscular activity and physiologic uptake. The scan extended from the skull base to the mid-thigh, with patients positioned supine and arms raised whenever feasible. A low-dose CT was first acquired for attenuation correction and anatomical localization (120 kV, 80–200 mAs, pitch 0.8–1.0, slice thickness 3–5 mm), followed by PET emission imaging at 2–3 min per bed position in 3D acquisition mode. Images were reconstructed using an OSEM algorithm with corrections for attenuation, scatter, random coincidences, and radioactive decay. Fused PET/CT images were reviewed on a dedicated Siemens syngo.via workstation.

### Diagnostic reference standard

The diagnostic reference standard was the final clinical diagnosis established through a combination of histopathological, microbiological, laboratory, and clinical evaluations. In cases with PET/CT-detected focal involvement, diagnosis was confirmed by targeted biopsy when clinically feasible. When PET/CT findings suggested specific inflammatory or rheumatologic conditions, disease-specific laboratory and rheumatologic investigations were performed to confirm the diagnosis. In cases without histopathological confirmation, the final diagnosis was determined based on comprehensive clinical assessment and follow-up findings.

### Definition of PET/CT contribution to disease diagnosis

- Cases in which PET/CT correctly identified the underlying cause of FUO or IUO were considered true positive.
- Cases in which no underlying disease was present and PET/CT was negative were considered true negative.
- Cases in which PET/CT suggested the presence of a disease but no underlying pathology was confirmed were considered false positive.
- Cases in which PET/CT did not indicate any disease but an underlying cause of FUO or IUO was subsequently identified were considered false negative.
- Patients in whom PET/CT was true positive or true negative were classified as cases in which PET/CT was useful.
- Patients in whom PET/CT was false positive or false negative were classified as cases in which PET/CT was not useful.

### Variables evaluated in the study

The following data were collected and analyzed from patient records:

1. *Demographic information*: age, sex, and body mass index (BMI).
2. *Comorbidities*: diabetes, hypertension, chronic kidney disease, chronic obstructive pulmonary disease, ischemic heart disease, cerebrovascular disease, malignancy, Parkinson’s disease, autoimmune disorders, presence of prosthetic heart valves or pacemakers, and immunosuppression status.
3. *Laboratory parameters*: white blood cell count, lymphocyte count, neutrophil count, hematocrit, platelet count, erythrocyte sedimentation rate, serum C-reactive protein (CRP), AST (SGOT), ALT (SGPT), alkaline phosphatase (ALP), gamma-glutamyl transferase (GGT), lactate dehydrogenase (LDH), ferritin, procalcitonin, D-dimer, and total bilirubin.
4. *Pre-PET/CT treatments*: use of steroids or antibiotic therapy prior to PET/CT imaging.

### Statistical analysis

#### General group comparisons

Patients in whom PET/CT was useful and those in whom it was not useful during follow-up were compared with respect to the variables described above. Statistical analyses were performed using SPSS software, version 16.0 (IBM SPSS Statistics, IBM Corp., Armonk, NY, USA). Categorical variables were expressed as frequencies and percentages. Continuous variables with normal distributions were presented as means ± standard deviations, whereas non-normally distributed variables were summarized as medians (minimum–maximum). Group comparisons were conducted using the Student’s *t*-test for normally distributed continuous variables and the Mann–Whitney *U* test for non-normally distributed variables. The chi-square test was used for comparisons of categorical variables. A two-tailed *p*-value < 0.05 was considered statistically significant.

#### Machine learning analysis

**Figure 1:**
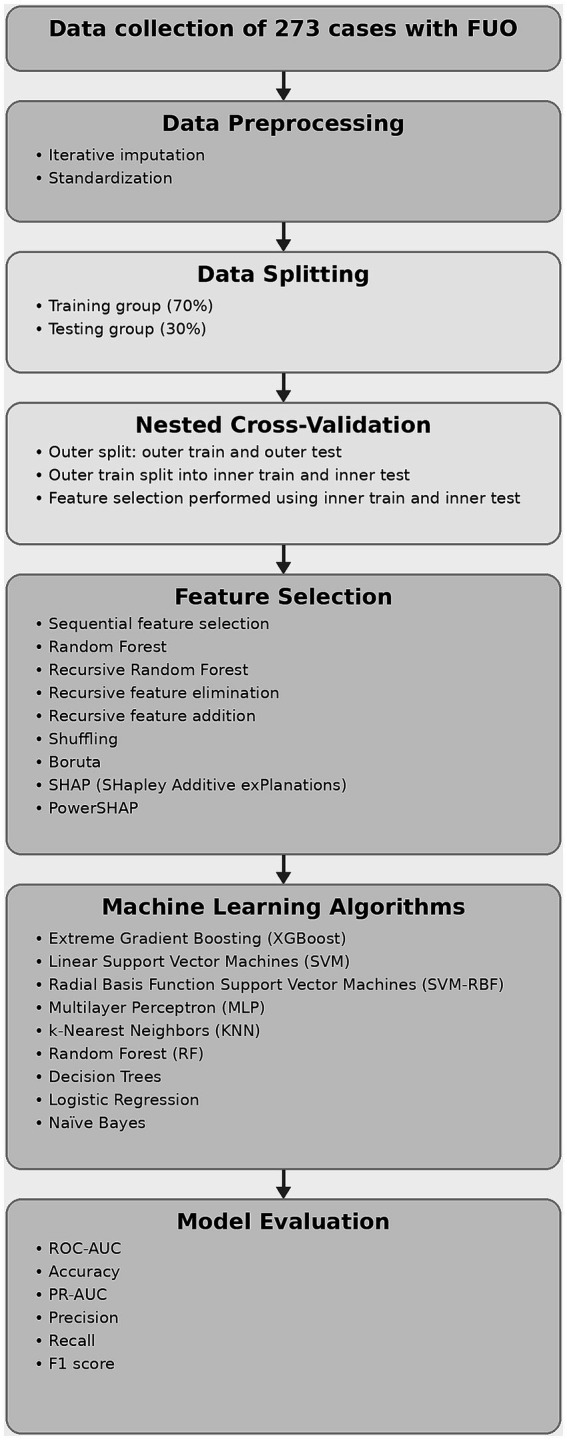
Overview of the machine learning framework for predicting PET/CT requirement in FUO patients.

Several machine learning methods were applied to predict the usefulness of PET/CT during follow-up. The overall workflow of the machine learning approach employed to predict PET/CT usefulness is illustrated in Figure 1.

##### Data preprocessing

Categorical variables were encoded using mapping and one-hot encoding, while continuous variables were standardized. Missing values were observed for several variables: symptom duration in 93 patients, erythrocyte sedimentation rate in 87 patients, procalcitonin in 94 patients, LDH in 103 patients, AST in 63 patients, ALT in 62 patients, and total bilirubin in 85 patients (Supplementary Table S1).

Missing values were imputed using the Iterative Imputer method (sklearn.impute.IterativeImputer), a multivariate regression-based approach that predicts missing values for each variable using the observed values of the others through an iterative series of regressions. By default, Random Forest Regressor were applied.

Finally, the dataset was split into training (70%) and testing (30%) subsets for machine learning analyses.

##### Feature selection

To reduce the number of independent variables and eliminate redundancy, several steps were applied. First, constant, quasi-constant, and duplicate variables were removed using the feature-engine package, with thresholds set at 80%, 0.998, and default settings, respectively.

Subsequently, multiple feature reduction techniques were evaluated to further refine the predictor set, including:

- Sequential feature selection.
- Random Forest.
- Recursive Random Forest.
- Recursive feature elimination.
- Recursive feature addition.
- Shuffling.
- Boruta.
- SHAP (SHapley Additive exPlanations).
- PowerSHAP.

To avoid information leakage and obtain an unbiased estimate of model performance, a nested evaluation strategy was implemented. The full dataset was first divided into an outer training set and an outer test set. The outer test set was completely isolated and used only once for final model evaluation, remaining untouched during feature selection, model training, and hyperparameter optimization.

Within the outer training set, an inner resampling framework was applied. The outer training data were split into inner training and inner validation (test) folds using cross-validation. Feature reduction methods were fitted exclusively on the inner training folds, and reduced feature sets were generated without access to outer test set. Each reduced feature set was then evaluated on the corresponding inner validation folds. Because of class imbalance, model performance during feature selection was primarily assessed using the precision–recall area under the curve (PR-AUC). Cross-validation across inner folds was used to obtain robust performance estimates.

The optimal feature selection method was determined based on a balance between predictive performance and model simplicity, selecting the approach that achieved the highest PR-AUC with the smallest number of features. The final reduced feature set identified by this procedure was subsequently used for model development.

##### Model development, evaluation, and explainability

Machine learning methods were implemented in Python to predict PET/CT usefulness in FUO/IUO patients. Binary classification models were developed using multiple algorithms, including:

- Extreme Gradient Boosting (XGBoost).
- Linear Support Vector Machines (SVM).
- Radial Basis Function Support Vector Machines (SVM-RBF).
- Multilayer Perceptron (MLP).
- k-Nearest Neighbors (KNN).
- Random Forest (RF).
- Decision Trees.
- Logistic Regression.
- Naïve Bayes.

Hyperparameter optimization was performed using GridSearchCV within the training data under a cross-validation framework. Separate optimization procedures were conducted using different scoring metrics (e.g., PR-AUC, ROC-AUC, and F1-score) to investigate the effect of metric-oriented model selection. Importantly, all hyperparameter tuning was restricted to the training data, and the outer test set was not used at any stage of model selection or optimization.

Following hyperparameter tuning, the best model obtained for each optimization metric was evaluated on the previously unseen outer test set. Model performance was assessed using accuracy, PR-AUC, ROC-AUC, precision, recall, and F1-score, allowing comprehensive comparison of models optimized under different criteria.

To address class imbalance, SMOTE (Synthetic Minority Over-sampling Technique) was applied only to the training data within the cross-validation pipeline, and class weights were adjusted where appropriate.

##### Performance metrics

Model performance was evaluated using confusion matrix–derived metrics, including precision, recall, and F1-score, as well as threshold-independent metrics such as ROC-AUC and PR-AUC.

To evaluate whether a simplified decision rule could achieve performance comparable to the full machine learning models, an additional analysis was performed using the top four variables derived from the most effective feature selection method. A logistic regression model was built using only these four variables, and its performance metrics were calculated. These results were then compared with those obtained from best machine learning models trained using the complete reduced feature set generated by the feature selection method, in order to assess the added value of using a larger set of variables and more complex algorithms.

To assess the impact of missing data imputation on model performance, all variables containing missing values were removed, and the full machine learning and feature selection pipeline was applied to this reduced, complete-case dataset. The performance metrics obtained from this complete-case analysis were then compared with those from the models trained on the imputed dataset to determine whether imputing missing values substantially affected the results.

## Results

A total of 273 patients were included in the study, comprising 151 males (55.3%) and 122 females (44.7%), with a mean age of 59 ± 16.9 years. During follow-up, PET/CT was found to be useful in 203 patients (74.4%). Table 1 summarizes baseline demographic, clinical, and laboratory characteristics according to PET/CT usefulness. Overall, most baseline variables were comparable between groups, including age, gender distribution, FUO/IUO status, symptom duration, and laboratory parameters. The rate of hospitalization during follow-up was significantly higher among patients in whom PET/CT was useful (74.4% vs. 48.6%, *p* = 0.0005). Weight loss and the presence of diabetes were more frequent in the non-useful PET/CT group, although these differences did not reach statistical significance (*p* = 0.068 and *p* = 0.06, respectively). PET/CT usefulness did not differ significantly between FUO and IUO patients.

**Table 1:** Comparison of demographic characteristics and laboratory parameters between patients with FUO/IUO in whom PET/CT was useful and those in whom it was not.

Variables		Patients in whom PET/CT was not useful (*n* = 70, 25.6%)	Patients in whom PET/CT was useful (*n* = 203, 74.4%)	*P*-value
Age (years)		66 ± 11.2	57 ± 23.0	0.309
Gender	Men	35 (50)	116 (57)	0.300
	Women	35 (50)	87 (42.9)	
Weight loss		15 (21.4)	67 (33)	0.068
Symptom duration		30 (20–36)	30 (16–36)	0.903
Immunosuppression		4 (5.7)	15 (7.4)	0.548
Follow up with hospitalization		34 (48.6)	151 (74.4)	0.0005
FUO/IUO status	FUO	37 (52.9)	128 (63.1)	0.132
	IUO	33 (75)	75 (36.9)	
Comorbidities	Diabetes	21 (30)	39 (19.2)	0.06
	Hypertension	21 (30)	44 (21.7)	0.158
	Chronic kidney disease	9 (12.9)	21 (10.3)	0.562
	Chronic obstructive pulmonary disease	5 (7.1)	13 (6.4)	0.785
	Ischemic heart disease	6 (8.6)	24 (11.8)	0.453
	Cerebrovascular disease	4 (5.7)	10 (4.9)	0.759
	Malignity	3 (4.3)	21 (10.3)	0.123
	Parkinson	2 (2.9)	5 (2.5)	1.000
	Autoimmune disorders	1 (2.3)	8 (6.7)	0.455
	Presence of prostetic heart valve and pacemaker	6 (8.6)	19 (9.4)	0.844
Patients who received antibiotic therapy prior to PET/CT imaging		11 (15.7)	30 (14.8)	0.850
Patients who received steroid therapy prior to PET/CT imaging		4 (5.7)	15 (7.4)	0.789
Symptoms	None	22 (31)	43 (21)	0.130
	Total body pain	23 (32)	102 (50)	
	Arthralgis	9 (12)	20 (9)	
	Shortness of breath	9 (12)	25 (12)	
	Abdominal pain	7 (10)	13 (6.4)	
Laboratory values	C-reactive protein (mg/L)	1.22 (4.32–215)	29 (4.32–217)	0.632
	Procalcitonin (ng/mL)	0.52 (0.14–5.020)	0.28 (0.02–7.920)	0.142
	D-Dimer (ng/mL)	1738 ± 119	1,548 ± 1,539	0.883
	Ferritin (ng/mL)	279 (46–907)	213 (9.66–1,438)	0.657
	Lymphocyte count (/×10^9^/L)	1.135 (1.010–3.160)	1.880 (0.930–4.720)	0.130
	Neutrophil count (/×10^9^/L)	4.697 ± 4.200	7.615 ± 4.100	0.923
	Leucocyte count (/×10^9^/L)	7.012 ± 4.800	9.694 ± 5.100	0.848
	Platelet (/×10^9^/L)	254 ± 131	269 ± 113	0.681
	Hematocrit	34 ± 3.7	37 ± 4.7	0.684
	Eritrosit sedimentation	67.5 (23–104)	35 (21–70)	0.479
	LDH	149 (111–631)	157 (111–201)	0.514
	ALP	64 (36–241)	83 (36–95)	0.730
	SGOT	22 (8–62)	21 (8–73)	0.691
	SGPT	54 (7–91)	20 (6–91)	0.516
	Total bilirubin	0.75 ± 0.53	0.38 ± 0.17	0.831

Regarding the final diagnoses established during follow-up, infection was identified as the most frequent underlying etiology, accounting for 45.8% (*n* = 125) of all cases. Rheumatologic diseases represented the second most common category, found in 22.0% (*n* = 60) of patients, followed by malignancy in 7.7% (*n* = 21) and other miscellaneous causes in 28 (10%) (Table 2).

**Table 2:** Grouping of patients according to established diagnoses in whom PET/CT was useful and not useful.

Final diagnosis	Patients in whom PET/CT was not useful (*n* = 70, 25.6%)	Patients in whom PET/CT was useful (*n* = 203, 74.4%)
No definite diagnosis	39 (55.7)	0 (0)
Malignity	0 (0)	21 (10.3)
Infection	19 (27.1)	106 (52.2)
Rheumatologic disease	10 (14.3)	50 (24.6)
Other disease	2 (2.9)	26 (12.8)

**Figure 2:**
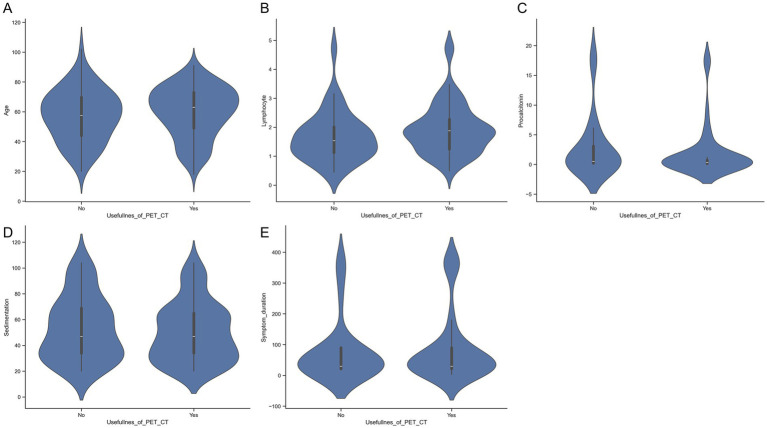
Violin plots showing the distribution of patient age (*p* = 0.309), symptom duration (*p* = 0.903), lymphocyte count (*p* = 0.130), procalcitonin level (*p* = 0.142), and erythrocyte sedimentation rate (*p* = 0.479) in FUO/IUO patients in whom PET/CT was useful versus not useful. The figure consists of five panels: **(A)** Age, **(B)** lymphocyte count, **(C)** procalcitonin level, **(D)** erythrocyte sedimentation rate, and **(E)** symptom duration.

Violin plots illustrating serum SGPT and lymphocyte count in patients with FUO/IUO in whom PET/CT was useful and not useful are shown in Figure 2.

In patients presenting with weight loss, malignancies and rheumatologic diseases were the most frequently identified disease groups, while in those with body pain, malignancies predominated (Table 3).

**Table 3:** Proportions of disease distribution according to the presence of whole-body pain and weight loss.

Variable	No disease (*n* = 39)	Malignancy (*n* = 21)	Infection (*n* = 125)	Rheumatologic diseases (*n* = 60)	Other (*n* = 28)	*p*-value
Weight loss, *n* (%)	8 (20.5)	10 (47.6)	24 (19.2)	25 (41.7)	15 (53.6)	0.0005
Body pain, *n* (%)	11 (28.2)	13 (61.9)	60 (48.0)	29 (48.3)	12 (42.9)	0.001

Values are presented as *n* (%).

**Figure 3:**
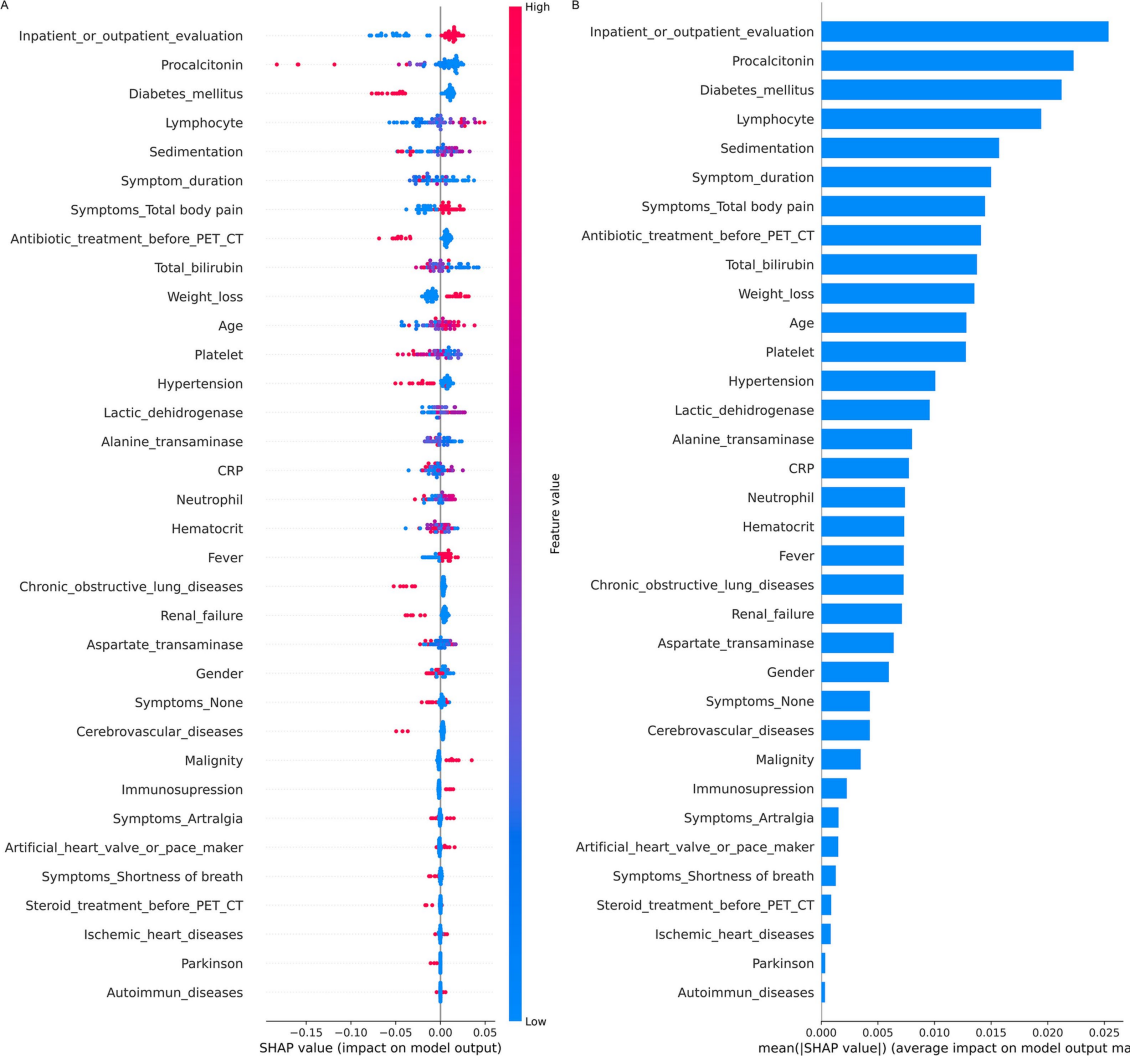
Ranked importance of variables for predicting the utility of PET/CT in patients with FUO: **(A)** SHAP summary plot showing the impact of each variable on model output. Each dot represents an individual patient, with color intensity indicating the actual feature value (red = high, blue = low). Variables are ordered by predictive significance. **(B)** Mean absolute SHAP values illustrating the average contribution of each variable to the model’s predictions.

In the dataset with imputed missing values, PowerSHAP, based on the tree algorithm, was identified as the best-performing feature reduction method. The PowerSHAP analysis suggested that lower procalcitonin and erythrocyte sedimentation rate levels, longer symptom duration, older age, generalized body pain, inpatient evaluation, and higher lymphocyte counts were associated with increased model-predicted PET/CT usefulness (Supplementary Table S2). Additionally, SHAP plots illustrating the feature importance of variables associated with PET/CT usefulness in FUO/IUO patients are presented in Figure 3.

**Figure 4:**
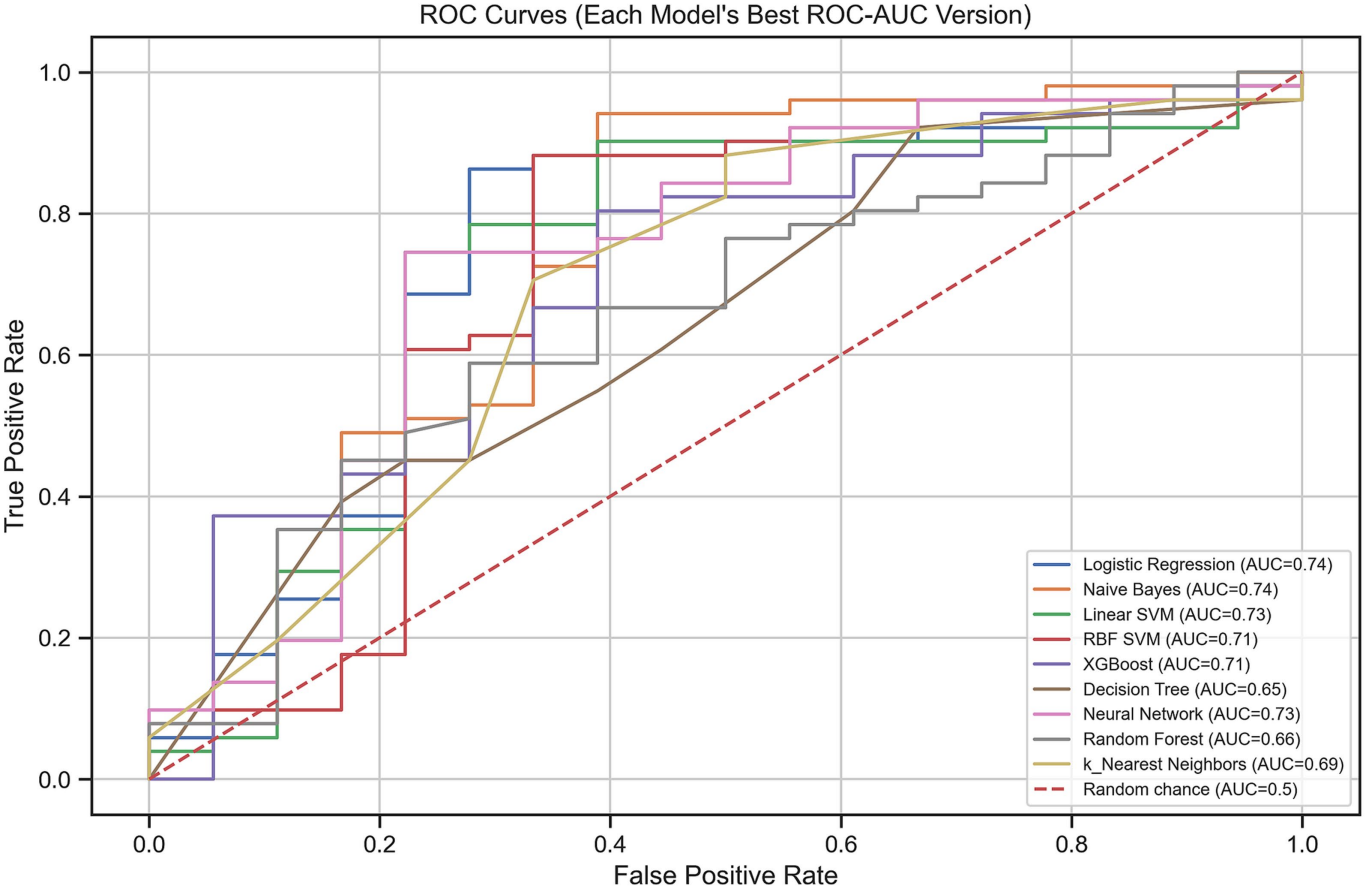
Receiver operating characteristic (ROC) curves of the machine learning models for predicting PET/CT usefulness in patients with FUO/IUO.

**Figure 5:**
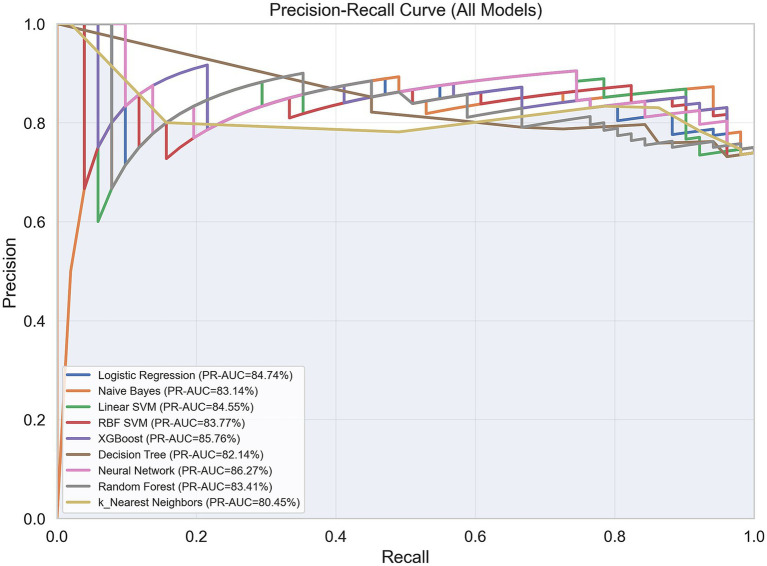
Precision–recall (PR) curves of the machine learning models for predicting PET/CT usefulness in patients with FUO/IUO.

**Figure 6:**
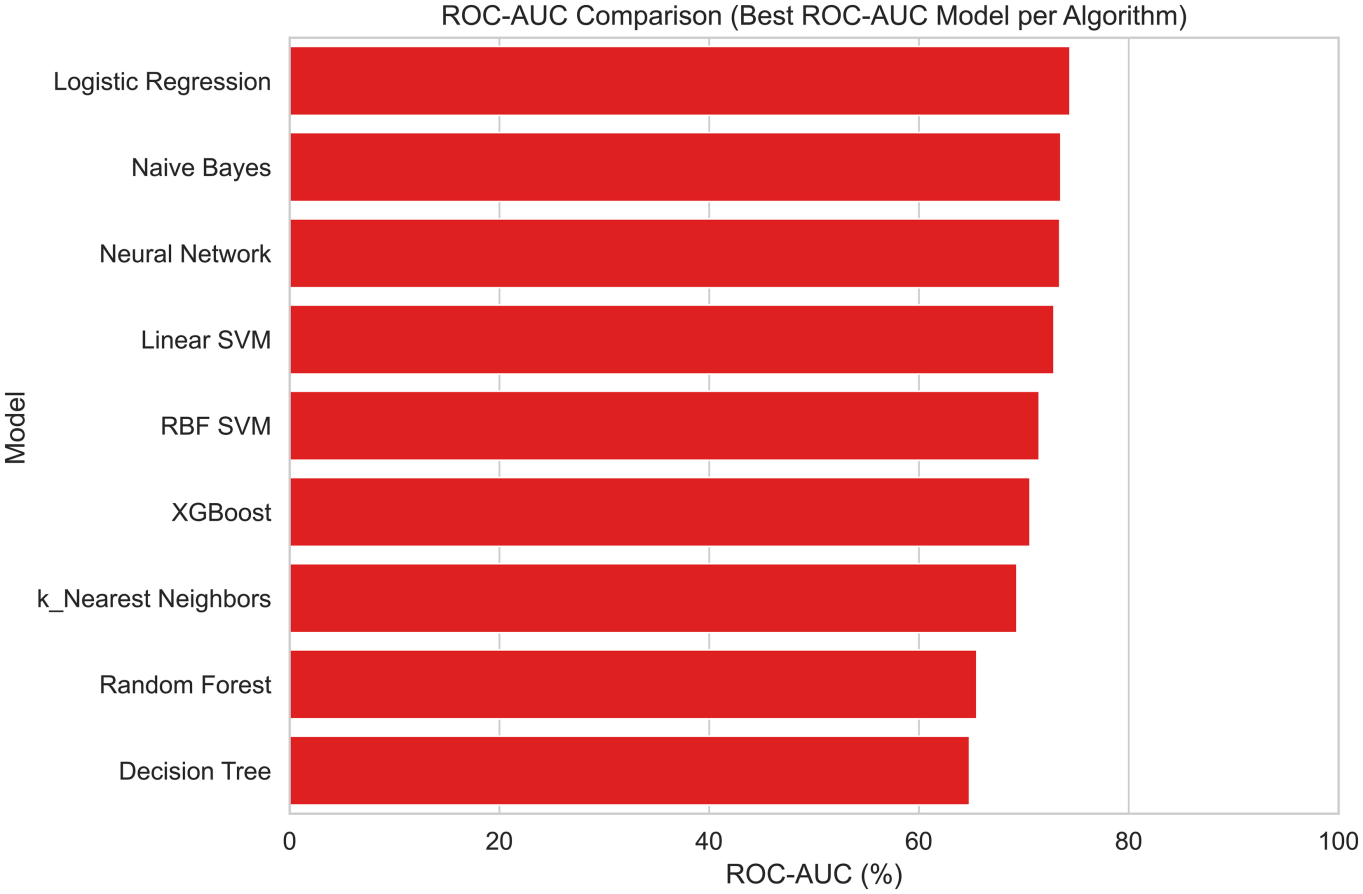
Comparison of model performance based on ROC-AUC values across machine learning algorithms (bar plot).

**Figure 7:**
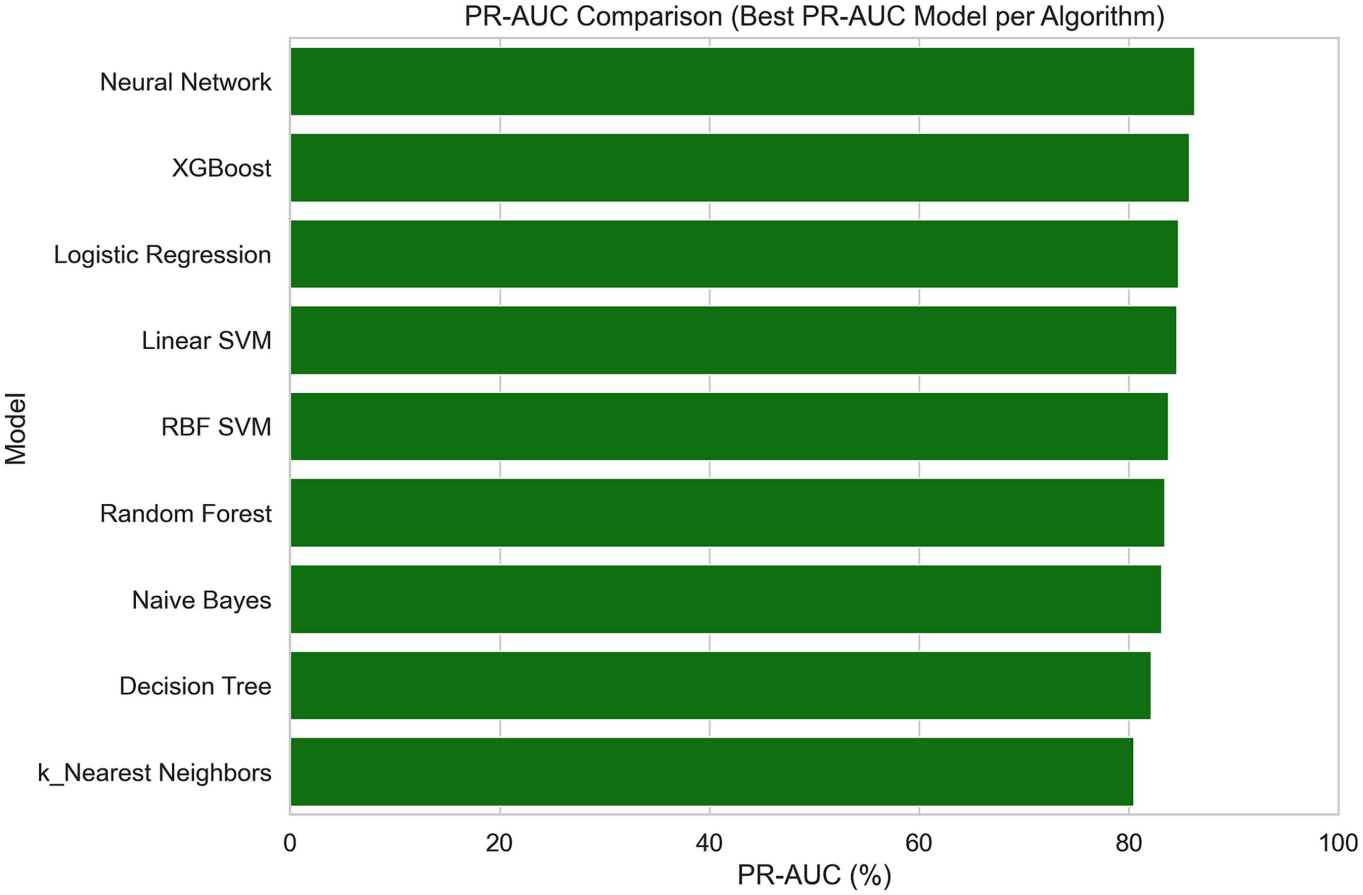
Comparison of model performance based on PR-AUC values across machine learning algorithms (bar plot).

**Figure 8:**
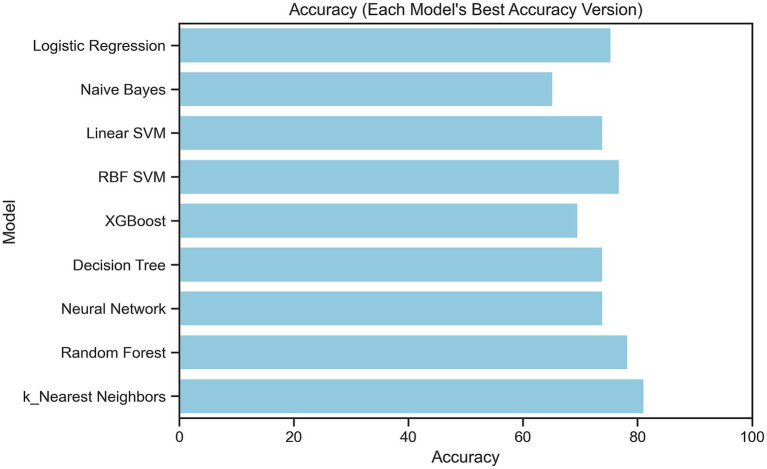
Comparison of model accuracy across machine learning algorithms (bar plot).

Several machine learning algorithms were applied to predict the utility of PET/CT in patients with FUO. Their performances are summarized in Table 4 and illustrated in Figures 4–8.

**Table 4:** Performance metrics [accuracy, area under the receiver operating characteristic curve (ROC-AUC), precision, recall, and F1-score] and tuning parameters of machine learning algorithms used to PET/CT usefulness in patients with FUO/IUO.

Algorithms	Tuned parameters	Accuracy	ROC-AUC	PR-AUC	Precision	Recall	F1
Logistic regression	PR-AUC: C = 0.001, maximum iterations = 5,000, penalty = L2, solve r = lbfgs Accuracy: C = 0.01, maximum iterations = 5,000, penalty = L2, solver = liblinear	75	74	84	85	86	83
Naïve bayes	—	65	73	83	84	64	73
K-nearest neighbors	PR-AUC: k = 13, Accuracy: k = 8	81	69	80	78	100	85
Linear support vector machine	PR-AUC: C = 8 Accuracy: C = 4	73	72	84	85	78	81
Radial basis function support vector macine	PR-AUC: C = 5, gamma = 0.1 Accuracy: C = 1, gamma = 10	76	71	83	80	100	85
Multilayer perceptron	PR-AUC: Activation = tanh, alpha = 0.02, hidden layer sizes = (5, 3), solver = adam Accuracy: Activation = logistic, alpha = 0.1, hidden layer sizes = (10, 10, 10), solver = sgd	73	73	86	85	100	85
XGBoost	PR-AUC: learning rate = 0.02, maximum depth = 6, n_estimators = 500, subsample = 0.8. Accuracy: learning rate = 0.02, maximum depth = 3, n_estimators = 2000, subsample = 1	69	70	85	84	78	77
Decision tree	PR-AUC: maximum depth = 8, minimum samples split = 28 Accuracy: maximum depth = 1, minimum samples split = 2	73	64	82	81	64	71
Random forest	PR-AUC: maximum feature = 2, maximum depth = 10, minimum sample split = 10, n_estimators = 10 Accuracy: maximum feature = 2, maximum depth = 8, minimum sample split = 2, subsample = 0.6, n_estimators = 500	78	65	83	82	94	86

All performance metrics are reported as percentages (%).

All algorithms performed well in terms of PR-AUC (>0.79), with the highest scores achieved by MLP and XGBoost, reaching PR-AUC values of 0.86 and 0.85, respectively. All algorithms except Naïve Bayes demonstrated good accuracy, and the highest accuracy values (0.81) were achieved by KNN. When both PR-AUC and accuracy were considered together, the best-performing models were MLP and Logistic Regression. Except for KNN all algorithms yielded high precision values (0.80–0.85), indicating a low rate of false positives. The algorithms with the highest recall values (all equal to 1.0), suggesting a low rate of false negatives, were KNN, MLP, and SVC RBF. All algorithms except Decision Tree demonstrated high F1-scores (0.73–0.86), indicating a good balance between precision and recall. Considering all metrics and models, Logistic Regression and MLP showed consistently strong performance. Logistic Regression achieved accuracy, ROC-AUC, PR-AUC, precision, recall, and F1-score values of 0.75, 0.74, 0.84, 0.85, 0.86, and 0.83, respectively, whereas MLP achieved 0.73, 0.73, 0.86, 0.85, 1.00, and 0.85.

Table 5 presents the performance comparison between the logistic regression model built using the top four variables from the optimal reduced feature set (i.e., the four highest-ranked features selected by PowerSHAP) and the multilayer perceptron model using the full reduced feature set.

**Table 5:** Performance comparison of a logistic regression model built using the top four features and a multilayer perceptron model using the full reduced feature set, both derived from PowerSHAP-selected features.

Metric	Logistic regression (4 variables)	Multilayer perceptron (6 variables)
ROC-AUC	70	70
PR-AUC	81	84
Accuracy	82	72
F1-score	88	98
Precision	85	84
Recall	94	100

All performance metrics are reported as percentages (%).

The logistic regression model achieved a ROC-AUC of 70%, PR-AUC of 81%, accuracy of 82%, F1-score of 88%, precision of 85%, and recall of 94%. The multilayer perceptron model demonstrated a similar ROC-AUC (70%) and a slightly higher PR-AUC (84%), with perfect recall (100%) and a higher F1-score (98%), but lower overall accuracy (72%) and comparable precision (84%). Overall, both models showed comparable discrimination performance, while the multilayer perceptron provided improved sensitivity at the expense of reduced accuracy.

**Figure 9:**
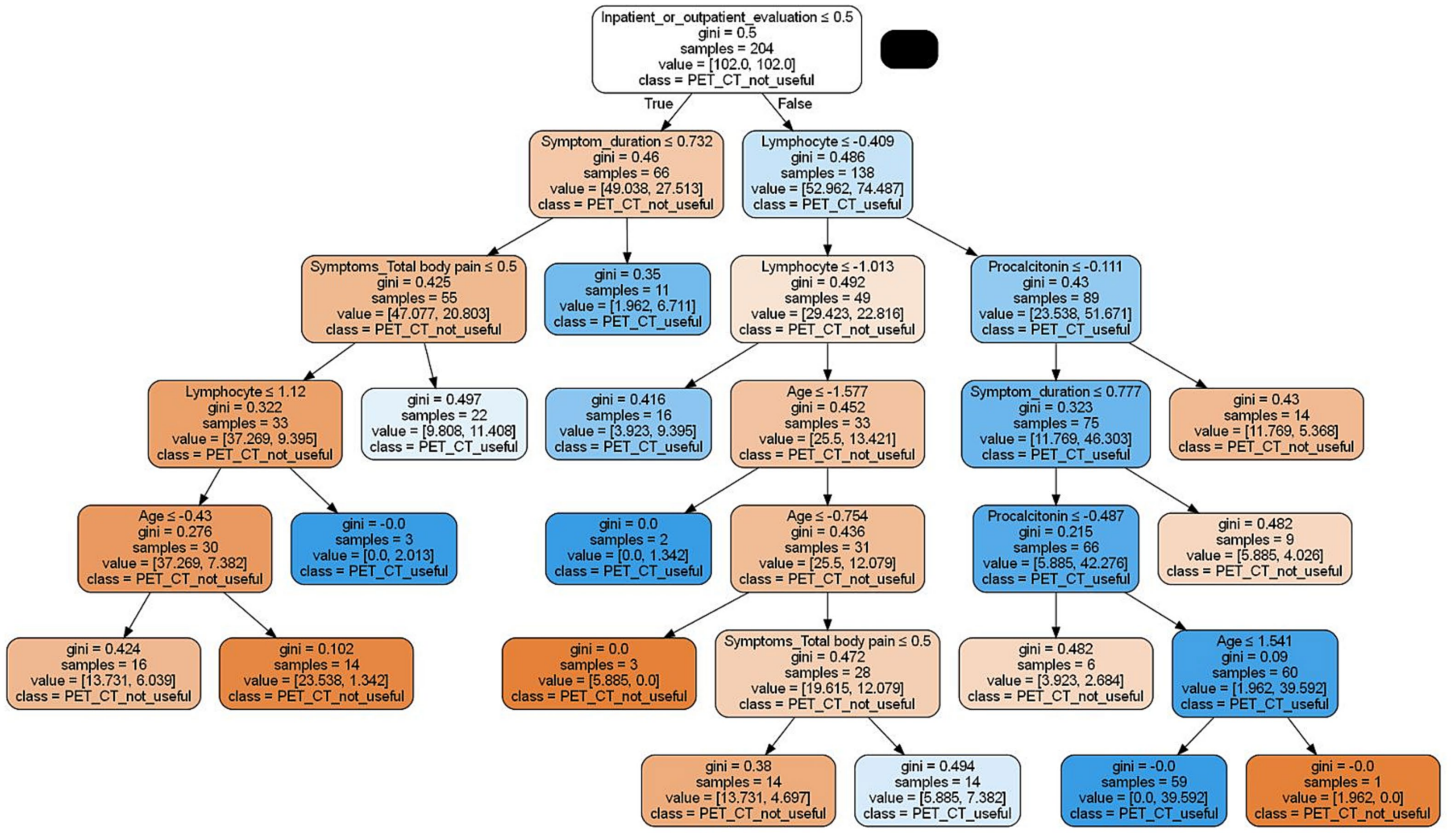
Decision tree algorithm for PET/CT selection in FUO/IUO patients.

The decision tree algorithm is illustrated in Figure 9. This model provides a structured approach that may help identify patients who could benefit from PET/CT imaging. The algorithm incorporates clinical and biochemical features, including symptom duration, presence of total body pain, lymphocyte count, age, and procalcitonin levels.

As described in the Materials and Methods section, after excluding variables with missing data, feature reduction was repeated using the remaining variables. The feature selection approach that achieved the highest PR-AUC with the smallest number of features was Shuffling, based on logistic regression (Supplementary Table S3).

The reduced feature set identified by this method included diabetes, renal failure, malignancy, weight loss, antibiotic use before PET, immunosuppression, inpatient/outpatient evaluation status, and the presence of a prosthetic valve or pacemaker.

Machine learning models constructed using these reduced features produced performance metrics that were comparable to those obtained from models trained on the imputed dataset (Supplementary Table S4). These findings indicate that missing data imputation did not substantially influence model performance or the overall study conclusions.

## Discussion

In this study, several ML algorithms, including LR, NB, linear and RBF SVM, KNN, MLP, CART, and RF, were applied to evaluate PET/CT usefulness in FUO/IUO patients.

Across all algorithms, PR-AUC values were consistently high (0.80–0.86), indicating strong discriminative ability and reliable model performance. High PR-AUC values across methods also suggest that the use of SMOTE oversampling and class weighting effectively mitigated the impact of class imbalance in the dataset. Moreover, PR-AUC is considered a more informative metric than ROC-AUC in imbalanced datasets, as it better reflects the model’s ability to identify positive cases under skewed class distributions (15).

When evaluating accuracy, all models except NB achieved satisfactory results. The relatively lower accuracy observed in this model may be explained by the correlated features within the dataset. In our experiments, the Naive Bayes (NB) classifier showed relatively poor accuracy compared to other models such as MLP, despite applying SMOTE for class balancing and adjusting class weights. Since NB assumes feature independence, the presence of correlated variables may cause the model to overcount shared information, leading to reduced classification performance (16). Nevertheless, NB can still capture meaningful patterns in the positive class, which may explain its comparatively high PR-AUC scores.

In terms of precision, which reflects the ability to avoid false positives, all models showed good performance, indicating that they effectively minimized incorrect positive classifications. The recall metric, which represents the sensitivity in detecting true positive cases, was highest for KNN, MLP, and RBF-SVM (recall = 1), indicating their strong ability to correctly identify all clinically positive PET/CT cases. The lowest recall was observed for NB and Decision Tree, both with a value of 0.64. When both PR-AUC and accuracy were considered, Logistic Regression (LR) and MLP demonstrated strong overall performance. While LR achieved slightly higher accuracy (0.75 vs. 0.73) and ROC-AUC (0.74 vs. 0.73), MLP slightly outperformed LR in PR-AUC (0.86 vs. 0.84) and F1-score (0.85 vs. 0.83), with a more pronounced advantage in recall (1.00 vs. 0.86), indicating that MLP captures more of the relevant positive cases. Notably, in the context of assessing PET/CT utility, this high recall is advantageous as it suggests that most patients with potential benefit are correctly identified, making recall a particularly relevant performance metric. The strong performance of the MLP model is likely due to its ability to capture complex interactions among variables (17), whereas the strong performance of LR suggests that the relationship between the target and predictors may be approximately linear.

To further assess the clinical relevance of model complexity, the performance of a simplified logistic regression model using the top four variables was compared with that of a multilayer perceptron model built using the full reduced feature set. Given the imbalance in the dataset, PR-AUC, recall, and F1-score provide a more informative evaluation than ROC-AUC. While both models showed similar precision (LR 85%, MLP 84%), the MLP model achieved higher PR-AUC (84% vs. 81%), F1-score (98% vs. 88%), and recall (100% vs. 94%), indicating better identification of patients with potential benefit. Logistic regression, on the other hand, maintained higher overall accuracy (82% vs. 72%), reflecting correct classification across the majority class. These results suggest that MLP may be preferable when prioritizing the detection of positive cases in PET/CT evaluation, whereas LR provides a simpler approach with robust overall accuracy. Notably, the strong performance of LR also indicates that a model capturing primarily linear relationships using a small number of top variables can achieve results comparable to a more complex approach like MLP that utilizes a larger feature set.

Previous studies, including a large Japanese retrospective analysis, have reported that FDG-PET/CT contributes more significantly to the diagnosis of IUO than FUO, with contributive findings observed in approximately 67% of IUO and 45% of FUO patients (4). This has been attributed to the higher prevalence of noninfectious inflammatory diseases (NIIDs), particularly rheumatologic and vasculitic disorders, among IUO cases, which often present with mild or absent fever, making them more likely to be classified as IUO and more readily detected by PET/CT due to their high metabolic activity (4). In our cohort, FDG-PET/CT was non-contributive in 37 FUO cases (52.9%) and 33 IUO cases (47.1%), and contributive in 128 FUO cases (63.1%) and 75 IUO cases (36.9%). The difference between FUO and IUO status was not statistically significant (*p* = 0.132), indicating a comparable diagnostic contribution of FDG-PET/CT across both groups. This suggests that FDG-PET/CT was equally useful for both FUO and IUO in our population. The absence of a higher diagnostic yield in IUO may be explained by the relatively lower proportion of rheumatologic diseases in our cohort, whereas infectious etiologies were more frequent. Given that FDG-PET/CT is particularly sensitive for detecting autoimmune and vasculitic inflammation, variations in the underlying disease spectrum likely influence its diagnostic performance. Overall, our findings highlight that the clinical utility of FDG-PET/CT in FUO and IUO depends largely on the distribution of underlying etiologies and may vary between populations. No significant difference was observed among patients who had received corticosteroids before PET/CT, which may be attributed to the relatively small number of steroid-treated cases. In our cohort, body pain was predominantly observed in patients with malignancies and rheumatologic diseases. These findings suggest that FUO patients presenting with body pain may have underlying conditions associated with intense systemic inflammation or high metabolic activity. Given that FDG-PET/CT detects regions of increased glucose metabolism, the presence of body pain may increase the likelihood of positive PET findings in malignancy and autoimmune disease, compared to infections or other less metabolically active etiologies. A review of the literature revealed that in a study evaluating 284 patients, widespread body pain and prolonged APTT were identified as the only factors associated with increased FDG-PET/CT utility in FUO cases (7). In another study involving 128 FUO patients, low hemoglobin levels and weight loss were reported as predictors of PET/CT usefulness (8). In our cohort, weight loss was more frequently observed in patients with malignancy, rheumatologic diseases, and other conditions. However, the “other etiologies” group—comprising less metabolically active conditions outside malignancy, rheumatologic diseases, and infections—was also relatively frequent. This may have contributed to a reduced overall utility of PET/CT in the subgroup of patients presenting with weight loss, as these conditions are less likely to produce detectable metabolic activity on FDG-PET/CT.

The decision tree offers an interpretable framework that may help guide clinicians in identifying FUO/IUO patients who could potentially benefit from PET/CT imaging. The model incorporates features such as symptom duration, total body pain, lymphocyte count, age, and procalcitonin levels.

Although elevated CRP, as a marker of increased inflammation, has been suggested in the literature to enhance FDG-PET/CT utility (9–11), our study did not support this association. Interestingly, in our cohort, PET/CT was even found to be useful in cases with low erythrocyte sedimentation rate and procalcitonin levels. While these findings are somewhat unexpected, they may be partially explained by a meta-analysis investigating vascular uptake in Takayasu arteritis, in which five studies reported a correlation between PET vascular activity and CRP levels, one study reported a weak correlation, and three studies found no correlation. These observations suggest that systemic inflammatory markers do not necessarily correlate with PET activity in all clinical contexts (18). Higher PET/CT utility in cases with low erythrocyte sedimentation rate and procalcitonin may be counterintuitive and could reflect the limited sample size, data variability, or other unmeasured confounding factors rather than a true mechanistic relationship. In one study, low lymphocyte counts were suggested to indicate viral infection, which could reduce FDG-PET/CT sensitivity (19, 20). Our findings were consistent with this pattern reported in the literature.

In our cohort, PET/CT appeared more useful in patients with a longer duration of symptoms. This observation may reflect the fact that, over time, disease processes progress and become more metabolically active, making pathological lesions easier to detect with PET/CT.

No studies in the literature have reported greater PET/CT utility in inpatients compared to outpatients. In our cohort, this observation may reflect that patients requiring hospitalization tend to have more severe disease, which could be associated with a higher burden of lesions detectable by PET/CT.

A limitation of our study is the relatively small sample size, which may increase the risk of overfitting and affect the stability of machine-learning models, as predictive performance generally improves with larger datasets. In addition, the study was conducted at a single center, which may limit the generalizability of the findings to other institutions or patient populations. Although missing values were imputed to allow complete-case analysis, imputation may not fully reflect real-world data availability and could influence model performance; however, sensitivity analyses suggested that imputation had minimal impact on the overall results. Furthermore, multiple ML algorithms were compared, and selection of the best-performing model may introduce model selection bias and optimistic performance estimates despite evaluation on an independent outer test set. Another limitation is the absence of an external validation cohort, which would provide a more robust assessment of generalizability. Finally, some predictors identified by the models lacked an immediately clear biological explanation, and therefore the findings should be interpreted cautiously until confirmed in prospective multicenter studies.

## Conclusion

In this study, multiple machine learning algorithms were evaluated to explore the potential diagnostic contribution of PET/CT in patients with fever or inflammation of unknown origin (FUO/IUO). The Multilayer Perceptron (MLP) and logistic regression (LR) models showed comparatively higher performance, suggesting that both non-linear and simpler interpretable approaches may capture relevant clinical and laboratory patterns. PowerSHAP analysis identified several variables associated with model predictions, including inpatient evaluation, generalized pain, procalcitonin level, erythrocyte sedimentation rate, age, lymphocyte count, and symptom duration; however, the biological plausibility of some predictors remains uncertain and should be interpreted cautiously. These findings should therefore be considered exploratory and hypothesis-generating rather than immediately applicable to clinical decision-making. Further prospective multicenter studies with external validation are required to confirm the robustness, generalizability, and clinical utility of these results before integration into routine clinical workflows.

## Data Availability

The original contributions presented in the study are included in the article/Supplementary material, further inquiries can be directed to the corresponding author.
