# Supplementary material for: Machine learning approach to determine the diagnostic value and predictive factors of PET/CT in FUO and IUO patients
Source: Front Med (Lausanne). 2026 Mar 16;13:1763501. doi: 10.3389/fmed.2026.1763501 (PMC13033511; doi:10.3389/fmed.2026.1763501)
Supplement: Supplementary file 1 [file Table_1.DOCX]

**Supplementary Table 1.** Missing data for key laboratory and clinical variables in the study cohort (n = 273).

| **Variable / Feature** | **Missing (n)** | **Missing (%)** |
| --- | --- | --- |
| **Symptom duration** | 93 | 34.1% |
| **Sedimentation** | 87 | 31.9% |
| **Procalcitonin** | 94 | 34.4% |
| **LDH** | 103 | 37.7% |
| **AST** | 63 | 23.1% |
| **ALT** | 62 | 22.7% |
| Total bilirubin | 85 | 31.1% |
