# Supplementary material for: Machine learning approach to determine the diagnostic value and predictive factors of PET/CT in FUO and IUO patients
Source: Front Med (Lausanne). 2026 Mar 16;13:1763501. doi: 10.3389/fmed.2026.1763501 (PMC13033511; doi:10.3389/fmed.2026.1763501)
Supplement: Supplementary file 2 [file Table_2.DOCX]

**Supplementary Table 2.** Performance of feature selection methods and the features they selected in the dataset with imputed missing values.

| Feature Selector | Model | Mean PR-AUC | Final Feature Set |
| --- | --- | --- | --- |
| Sequential Feature Selection | TREE | 0.8019 | Age, Symptom duration, Platelet, Procalcitonin, Fever, Symptoms None, Total body pain, Inpatient or outpatient evaluation, Lymphocyte, Gender, Hematocrit, Sedimentation, Shortness of breath, CRP, Chronic obstructive lung diseases, Antibiotic treatment before PET CT, Neutrophil, Arthralgia, Hypertension, Lactic dehidrogenase, Autoimmun diseases, Parkinson, Steroid treatment before PET CT |
| Lasso Logistic | LOGISTIC | 0.8604 | Neutrophil, Hematocrit, CRP, Procalcitonin, Alanine transaminase, Total bilirubin, Total body pain, Diabetes mellitus, Hypertension, Cerebrovascular diseases, Malignity, Weight loss, Inpatient or outpatient evaluation, Lymphocyte, Platelet, Aspartate transaminase, Fever, Renal failure |
| Recursive Random Forest | TREE | 0.7995 | Age, Sedimentation, Symptom duration, Procalcitonin, Lactic dehidrogenase, Lymphocyte, CRP, Hematocrit, Aspartate transaminase, Platelet, Alanine transaminase, Total bilirubin, Neutrophil, Diabetes mellitus, Fever, Renal failure, Gender, Inpatient or outpatient evaluation, Antibiotic treatment before PET CT, Total body pain, Arthralgia, Weight loss, Hypertension, No Symptoms, Cerebrovascular diseases, Shortness of breath, Chronic obstructive lung diseases, Ischemic heart diseases, Artificial heart valve or pace maker |
| Recursive Feature Elimination | TREE | 0.7789 | Age, Symptom duration, Neutrophil, Lymphocyte, CRP, Sedimentation, Procalcitonin, Alanine transaminase, Artralgia, No symptoms , Total body pain, Gender, Diabetes mellitus, Hypertension, Renal failure, Chronic obstructive lung diseases, Ischemic heart diseases, Malignity, Weight loss, Antibiotic treatment before PET CT, Steroid treatment before PET CT, Artificial heart valve or pace maker, Inpatient or outpatient evaluation, Platelet, Total bilirubin, Cerebrovascular diseases, Parkinson, Immunosupression, Fever |
| Recursive Feature Elimination | LOGISTIC | 0.8096 | Procalcitonin, Weight loss, Antibiotic treatment before PET CT, Inpatient or outpatient evaluation, CRP, Total body pain |
| Recursive Addition | TREE | 0.7848 | Procalcitonin, Age, Symptom duration, Symptoms Total body pain, Total bilirubin, Platelet, Weight loss, Renal failure, Symptoms Shortness of breath, Symptoms None, Fever, Malignity, Lymphocyte, Hypertension, Lactic dehidrogenase, Neutrophil, Alanine transaminase, Sedimentation, Hematocrit |
| Recursive Addition | LOGISTIC | 0.8466 | Diabetes mellitus, Inpatient or outpatient evaluation, Weight loss, Fever, Total body pain, Malignity, Parkinson, Cerebrovascular diseases, Procalcitonin, Gender, Steroid treatment before PET CT, Lymphocyte, Immunosupression, Artificial heart valve or pace maker, Antibiotic treatment before PET CT, Platelet, Hypertension, Renal failure, Neutrophil, Total bilirubin, CRP, Symptoms Shortness of breath, Alanine transaminase, Aspartate transaminase, Ischemic heart diseases, No symptoms, Autoimmun diseases |
| Shuffling | TREE | 0.7998 | Symptom duration, Procalcitonin, Arthralgia, Symptoms None, Hypertension, Cerebrovascular diseases, Antibiotic treatment before PET CT, Artificial heart valve or pace maker, Inpatient or outpatient evaluation, Diabetes mellitus, Ischemic heart diseases, Malignity, Parkinson, Autoimmun diseases, Weight loss, Steroid treatment before PET CT, Immunosupression, Lymphocyte |
| Shuffling | LOGISTIC | 0.8307 | Age, Lymphocyte, Hematocrit, Procalcitonin, Lactic dehidrogenase, Alanine transaminase, No Symptoms, Total body pain, Diabetes mellitus, Cerebrovascular diseases, Malignity, Immunosupression, Platelet, Renal failure, Weight loss, Inpatient or outpatient evaluation |
| PowerSHAP | LOGISTIC | 0.7918 | Inpatient or outpatient evaluation, Procalcitonin, Symptom duration, Sedimentation, Age, Lymphocyte, CRP, Fever, Weight loss, Total body pain |
| PowerSHAP | TREE | 0.8063 | Inpatient or outpatient evaluation, Procalcitonin, Symptom duration, Sedimentation, Age, Lymphocyte, Total body pain |
| SHAP | TREE | 0.8270 | Inpatient or outpatient evaluation, Age, Lymphocyte, Symptom duration, Procalcitonin, Total body pain, Sedimentation, Diabetes mellitus, Weight loss, Lactic dehidrogenase, Fever, CRP, Alanine transaminase, No symptoms, Total bilirubin, Hematocrit, Aspartate transaminase, Neutrophil, Renal failure, Platelet |
