# Supplementary material for: Machine learning approach to determine the diagnostic value and predictive factors of PET/CT in FUO and IUO patients
Source: Front Med (Lausanne). 2026 Mar 16;13:1763501. doi: 10.3389/fmed.2026.1763501 (PMC13033511; doi:10.3389/fmed.2026.1763501)
Supplement: Supplementary file 3 [file Table_3.DOCX]

**Supplementary Table 3.** Performance of feature selection methods and the features they selected after removing all columns with missing values

| **Feature Selector** | **Model** | **Mean PR-AUC** | **Final Feature Set** |
| --- | --- | --- | --- |
| Sequential Feature Selection | TREE | 0.7748 | Age, Lymphocyte, Platelet, Total body pain, CRP, Gender, Inpatient or outpatient evaluation, Fever, Hematocrit, Artralji, Cerebrovascular diseases, No symptom, Renal failure, Chronic obstructive lung diseases, Weight loss, Antibiotic treatment before PET CT, Ischemic heart diseases, Diabetes mellitus, Hypertension, Neutrophil, Shortness of breath, Parkinson, Artificial heart valve or pace maker |
| Lasso Logistic | LOGISTIC | 0.8582 | Neutrophil, Lymphocyte, Hematocrit, CRP, Total body pain, Gender, Diabetes mellitus, Hypertension, Cerebrovascular diseases, Malignity, Weight loss, Inpatient or outpatient evaluation, Platelet, Fever, Renal failure |
| Recursive Random Forest | TREE | 0.8167 | Age, Lymphocyte, CRP, Hematocrit, Platelet, Neutrophil, Diabetes mellitus, Gender, Fever, Antibiotic treatment before PET CT, Renal failure, Inpatient or outpatient evaluation, Hypertension, Weight loss, No symptom, Total body pain, Artralji, Shortness of breath, Cerebrovascular diseases, Ischemic heart diseases, Artificial heart valve or pace maker, Malignity, Chronic obstructive lung diseases |
| Recursive Feature Elimination | TREE | 0.8086 | Age, Platelet, Hematocrit, Fever, No symptom, Shortness of breath, Gender, Hypertension, Renal failure, Chronic obstructive lung diseases, Ischemic heart diseases, Cerebrovascular diseases, Malignity, Parkinson, Weight loss, Antibiotic treatment before PET CT, Steroid treatment before PET CT, Artificial heart valve or pace maker, Immunosupression, Inpatient or outpatient evaluation, Lymphocyte, Artralji, Total body pain, Diabetes mellitus |
| Recursive Feature Elimination | LOGISTIC | 0.8245 | CRP, Diabetes mellitus, Malignity, Weight loss, Antibiotic treatment before PET CT, Inpatient or outpatient evaluation, Lymphocyte |
| Recursive Addition | TREE | 0.8216 | Age, Diabetes mellitus, Antibiotic treatment before PET CT, Inpatient or outpatient evaluation, Platelet, Weight loss, Immunosupression, Total body pain, Lymphocyte, Steroid treatment before PET CT, Malignity, Gender, Artificial heart valve or pace maker, Neutrophil, Fever, Shortness of breath, Hypertension, Autoimmun diseases, Cerebrovascular diseases, Hematocrit |
| Recursive Addition | LOGISTIC | 0.8392 | Total body pain, Weight loss, Inpatient or outpatient evaluation, Immunosupression, Diabetes mellitus, Fever, Lymphocyte, Cerebrovascular diseases, Renal failure, Malignity, Antibiotic treatment before PET CT, Ischemic heart diseases, No symptom, Parkinson, Steroid treatment before PET CT, Platelet, Chronic obstructive lung diseases |
| Shuffling | TREE | 0.7886 | Age, Lymphocyte, Platelet, Parkinson, Weight loss, Inpatient or outpatient evaluation, Artralji, Diabetes mellitus, Chronic obstructive lung diseases, Ischemic heart diseases, Artificial heart valve or pace maker, Immunosupression |
| Shuffling | LOGISTIC | 0.8642 | Diabetes mellitus, Renal failure, Malignity, Weight loss, Antibiotic treatment before PET CT, Immunosupression, Inpatient or outpatient evaluation, Artificial heart valve or pace maker |
| PowerSHAP | LOGISTIC | 0.8007 | Inpatient or outpatient evaluation, Lymphocyte, Age, CRP, Neutrophil, Platelet, Weight loss, Fever |
| PowerSHAP | TREE | 0.7834 | Inpatient or outpatient evaluation, Lymphocyte, Age, CRP, Neutrophil, Platelet, Weight loss, Fever, Total body pain |
| SHAP | TREE | 0.8580 | Inpatient or outpatient evaluation, Age, Weight loss, Diabetes mellitus, Platelet, Fever, Lymphocyte, Neutrophil, CRP, Total body pain, No symptom, Hematocrit, Renal failure, Gender, Malignity |
