# Supplementary material for: Machine learning approach to determine the diagnostic value and predictive factors of PET/CT in FUO and IUO patients
Source: Front Med (Lausanne). 2026 Mar 16;13:1763501. doi: 10.3389/fmed.2026.1763501 (PMC13033511; doi:10.3389/fmed.2026.1763501)
Supplement: Supplementary file 4 [file Table_4.DOCX]

**Supplementary Table 4.** Performance metrics of main machine learning models applied to the final reduced feature set obtained after removing columns with missing data.

| **Algorithms** | **ROC-AUC** | **PR-AUC** | **Accuracy** | **F1** | **Precision** | **recall** |
| --- | --- | --- | --- | --- | --- | --- |
| **Logistic regression** | 60 | 78 | 78 | 85 | 83 | 86 |
| **Naïve Bayes** | 60 | 77 | 34 | 34 | 66 | 23 |
| **K-Nearest Neighbors** | 63 | 79 | 76 | 86 | 76 | 98 |
| **Linear Support Vector Machine** | 64 | 79 | 65 | 73 | 82 | 66 |
| **Radial Basis Function Support Vector Macine** | 64 | 79 | 73 | 82 | 82 | 82 |
| **Multilayer Perceptron** | 66 | 79 | 73 | 85 | 73 | 100 |
| **XGBoost** | 70 | 76 | 63 | 73 | 85 | 100 |
| **Decision Tree** | 56 | 75 | 71 | 79 | 82 | 76 |
| **Random Forest** | 61 | 76 | 68 | 76 | 82 | 70 |

All performance metrics are reported as percentages (%).
